# Genome-Wide Identification, Evolutionary Expansion, and Expression Profile of Homeodomain-Leucine Zipper Gene Family in Poplar (*Populus trichocarpa*)

**DOI:** 10.1371/journal.pone.0031149

**Published:** 2012-02-16

**Authors:** Ruibo Hu, Xiaoyuan Chi, Guohua Chai, Yingzhen Kong, Guo He, Xiaoyu Wang, Dachuan Shi, Dongyuan Zhang, Gongke Zhou

**Affiliations:** 1 CAS Key Laboratory of Biofuels, Shandong Provincial Key Laboratory of Energy Genetics, Qingdao Institute of BioEnergy and BioProcess Technology, Chinese Academy of Sciences, Qingdao, Shandong, People's Republic of China; 2 Shandong Peanut Research Institute, Qingdao, Shandong, People's Republic of China; 3 Complex Carbohydrate Research Center, University of Georgia, Athens, Georgia, United States of America; University of Toronto, Canada

## Abstract

**Background:**

Homeodomain-leucine zipper (HD-ZIP) proteins are plant-specific transcriptional factors known to play crucial roles in plant development. Although sequence phylogeny analysis of *Populus* HD-ZIPs was carried out in a previous study, no systematic analysis incorporating genome organization, gene structure, and expression compendium has been conducted in model tree species *Populus* thus far.

**Principal Findings:**

In this study, a comprehensive analysis of *Populus* HD-ZIP gene family was performed. Sixty-three full-length HD-ZIP genes were found in *Populus* genome. These *Populus* HD-ZIP genes were phylogenetically clustered into four distinct subfamilies (HD-ZIP I–IV) and predominately distributed across 17 linkage groups (LG). Fifty genes from 25 *Populus* paralogous pairs were located in the duplicated blocks of *Populus* genome and then preferentially retained during the sequential evolutionary courses. Genomic organization analyses indicated that purifying selection has played a pivotal role in the retention and maintenance of *Populus* HD-ZIP gene family. Microarray analysis has shown that 21 *Populus* paralogous pairs have been differentially expressed across different tissues and under various stresses, with five paralogous pairs showing nearly identical expression patterns, 13 paralogous pairs being partially redundant and three paralogous pairs diversifying significantly. Quantitative real-time RT-PCR (qRT-PCR) analysis performed on 16 selected *Populus* HD-ZIP genes in different tissues and under both drought and salinity stresses confirms their tissue-specific and stress-inducible expression patterns.

**Conclusions:**

Genomic organizations indicated that segmental duplications contributed significantly to the expansion of *Populus* HD-ZIP gene family. Exon/intron organization and conserved motif composition of *Populus* HD-ZIPs are highly conservative in the same subfamily, suggesting the members in the same subfamilies may also have conservative functionalities. Microarray and qRT-PCR analyses showed that 89% (56 out of 63) of *Populus* HD-ZIPs were duplicate genes that might have been retained by substantial subfunctionalization. Taken together, these observations may lay the foundation for future functional analysis of *Populus* HD-ZIP genes to unravel their biological roles.

## Introduction

Homeodomain (HD) proteins play fundamental roles in a diverse set of plant developmental processes, from pattern formation to cell type specification [1]. HD proteins constitute a large family of transcription factors with the HD DNA-binding domain at N-termini. In plants, HD proteins can be classified into 14 distinct families based on the sequence similarity of HD domains and their unique codomains [2]. Homeodomain-leucine zipper (HD-ZIP) genes are the most abundant group of HD genes in plants but no HD-ZIP genes present in other eukaryotes. Unique features of HD-ZIP proteins are the presence of a HD domain and an adjacent Leucine Zipper (LZ) motif [3]. The HD domain is responsible for specific DNA binding, whereas the LZ motif mediates protein dimerization [4], [5], [6], [7]. Based on the DNA-binding specificities, additional conserved motifs and their physiological functions, HD-ZIP genes are divided into four subfamilies (HD-ZIP I, II, III and IV) [7], [8].

*Arabidopsis* HD-ZIP I subfamily has 17 members (*ATHB1/HAT5*, *ATHB3/HAT7*, *ATHB5–7*, *ATHB12*, *ATHB13*, *ATHB16*, *ATHB20–23*, *ATHB40*, *ATHB51–54*) [9]. *Arabidopsis* HD-ZIP I genes are not only responsive to sugar signaling, abscisic acid (ABA) signaling and abiotic stresses, but also critical to plant embryogenesis and de-etiolation. *ATHB13* are potentially regulating sugar signaling [10] and *ATHB5*, *ATHB6*, *ATHB7*, and *ATHB12* have been proposed to participate in ABA-dependent and abiotic stress responses [11], [12], [13], [14], [15], [16], of which *ATHB6* is a crucial regulator in the ABA signaling pathway [17]. *ATHB16* participates not only in blue-light signaling but also in leaf cell expansion [18], whereas *ATHB52* regulates photomorphogenesis and de-etiolation [9]. Other HD-ZIP I genes, such as *ATHB1*, *ATHB3*, *ATHB20*, and *ATHB23* are involved in cotyledon and leaf development [19], [20] and *ATHB1* also responses to de-etiolation of dark-grown seedlings [19]. Recent progress shows that white spruce (*Picea glauca*) HD-ZIP I proteins also participate in ABA responses [21].

*Arabidopsis* HD-ZIP II subfamily consists of nine members (*ATHB2/HAT4*, *ATHB4*, *HAT1–HAT3*, *HAT9*, *HAT14*, *HAT17*, and *HAT22*) [22]. All nine members have a cellular redox status perceptive CPSCE (Cys, Pro, Ser, Cys, and Glu) motif at the downstream of LZ motif [23] and most of these genes are mainly respond to light, shading and auxin as revealed by genetic and biochemical analyses [24], [25], [26], [27]. Both *ATHB2/HAT4* and *HAT2* regulate auxin-mediated morphogenesis in *Arabidopsis*. *ATHB2/HAT4* mediates red/far-red light effects on leaf cell expansion and shade avoidance intrigued by three distinct phytochromes [24], [27]. *HAT2* is also auxin-inducible in *Aradibopsis* seedlings [26]. Ectopic expression of *HAT2* in *Arabidopsis*, consistent with the typical phenotypes of other auxin-overproducing mutants, produces a variety of phenotypic deviations with long hypocotyls, epinastic cotyledons, long petioles and small leaves [25]. Research on sunflowers also shows that their HD-ZIP II genes act as developmental regulators in response to illumination [28].

*Arabidopsis* HD-ZIP III subfamily comprises of only five genes, *PHABULOSA(PHB)/ATHB14*, *PHAVOLUTA(PHV)/ATHB9*, *REVOLUTA (REV)/INTERFASCICULAR FIBERLESS1(IFL1)*, *ATHB8*, and *CORONA(CNA)/ATHB15/INCURVATA4 (ICU4)* [29], but they are the key developmental regulators of *Arabidopsis* apical embryo patterning, shoot meristem formation, vascular differentiation, organ polarity determination, as well as auxin transportation [29], [30], [31], [32], [33], [34], [35], [36]. Members of *Arabidopsis* HD-Zip III subfamily have an N-terminal putative steroid/lipid-binding START (STeroidogenic Acute Regulatory protein related lipid Transfer) domain, followed by an adjacent conserved SAD (START-adjacent) domain [37], [38], and a C-terminal PAS-related MEKHLA domain that potentially involves in oxygen redox perception and light signaling [39]. Three closely related *Arabidopsis* HD-ZIP III genes, *REV/IFL1*, *PHB/ATHB9*, and *PHV/ATHB14*, act antagonistically with *KANADI* to regulate the establishment of apical meristem, vascular pattern, and adaxial domains in lateral organs [31], [33], [35], [36]. These three HD-ZIP III genes have partially overlapping roles as the single mutants have only minor or even no apparent defects in interfascicular fiber formation as well as floral and lateral meristem initiation [29], but the *phb rev* and *phv reb* double mutants enhance these defects [29] and the *phb phv rev* triple mutants have no apical meristem but substituted by a single radialized cotyledon [29], [31]. Another set of two closely related HD-ZIP III members, *ATHB8* and *CNA/ATHB15/ICU4*, act as regulators of vascular development [30], [32], [34], as *athb8* mutants bear no detectable phenotypes but constitutive expression of *ATHB8* triggers premature initiations of the secondary growth in xylem cells that eventually leads to the ectopic proliferation of xylem cells [30]. Additional evidences show that *ATHB8* also regulates the initiation of procambial development, vein patterning and differentiation [40]. Genetic evidence also show that *CNA/ATHB15/ICU4* gene presumably acts as a negative regulator of procambial cell identity or proliferation because ectopic expression of a miRNA-resistant *CNA/ATHB15/ICU4* results in moderate dwarfing stature with drastic reduction in xylem and lignified interfascicular tissues, whereas antisense *ATHB15* transformants are severely dwarfed with expanded xylem and interfascicular fibers, and ectopic lignified pith [41]. Some other evidences show that the HD-Zip III family transcripts are also specifically targeted and thus negatively regulated by MiRNA165/166 [31], [33], [41], [42], [43], [44], [45].

In *Arabidopsis*, HD-ZIP IV (also known as HD-GL2) constitutes a large subfamily of genes composed of 16 members: *GLABRA2(GL2)/ATHB10*, *ARABIDOPSIS THALIANA MERISTEM LAYER1(ATML1)*, *ANTHOCYANINLESS2(ANL2)*, *PROTODERMAL FACTOR2 (PDF2)*, *HOMEODOMAIN GLABROUS 1(HDG1)-HDG5*, *HDG6/FWA, and HDG7–HDG12* [46]. HD-ZIP IV proteins have the similar domain arrangements to those of HD-ZIP III members but only lack of the C-terminal MEHKLA domain, suggesting that class III and IV HD-Zip gene families may share a common ancestor [7], [38]. Genetic analysis shows that HD-ZIP IV proteins play crucial roles in epidermal cell differentiation, trichome formation, root development and anthocyanin accumulation. Three HD-ZIP IV genes, *GL2/ATHB10*, *ATML1*, and *PDF2*, appear to determinate the fate of epidermal layer cells [47], [48], [49], [50], [51]. The *GL2* determines trichome outgrowth in shoot epidermal cells as well as root hair cell specification as mutant *gl2* has impaired trichome and defected root hair [47], [48], [52]. Loss-of-function mutants of either *atml1* or *pdf2* mutants also show severe defects in shoot epidermal cell differentiation, and the *atml1 pdf2* double mutants fail to differentiate into protoderm during embryogenesis and thus are embryonic lethal [51]. Another set of HD-ZIP IV genes, *HDG11* and *HDG12*, repress trichome outgrowth. *hdg11* single mutant leads to excessive trichome branching and *hdg12* single mutant has no trichome, while the *hdg11 hdg12* double mutants have more excessive trichome branching [46]. *ANL2* regulates anthocyanin accumulation in leaf sub-epidermal layer as well as cell identity in root [53].

Compared to the largely investigated functions of *Arabidopsis* HD-ZIPs, only two *Populus* HD-ZIP genes (*POPREVOLUTA* and *POPCORONA*) have recently been characterized. These two HD-ZIP III genes are largely involved in regulating cell differentiation during secondary growth. Misexpression of *POPREVOLUTA* (*PRE*, a *Populus* orthologue of *REV/IFL1*) induces cambium initiation in abnormal positions and eventually causes patterning defects in derived secondary vascular tissues, even, to the extent of complete polarity reversals [54]. Overexpression of a miRNA-resistant *POPCORONA* (*PCN*, a *Populus CNA/ATHB15/ICU4* orthologue) shows delayed lignification of xylem and phloem fibers during secondary growth, whereas synthetic miRNA knockdown of *PCN* has abnormal lignification in pith cells [55].

Recently, a complete survey and classification of HD genes in ten different plant species from disparate evolutionary groups has already been carried out [2], however, only sequence phylogeny analyses of *Populus* HD-ZIPs are performed in the previous study and no detailed systematic analysis including genome organization, gene structure and expression compendium has been conducted. In this study, we first performed a genome-wide identification of HD-ZIP genes in *Populus* to reveal an expanded HD-ZIP family with totally 63 members, and then analyzed the sequence phylogeny, genome organization, gene structure, conserved motifs and expression profiling of these 63 genes. Furthermore, a thorough comparative analysis of *Populus* HD-ZIP genes to those from seven other plant species was performed. We finally examined the gene expression patterns of 16 *Populus* HD-ZIP genes to verify the evolution origins of *Populus* HD-ZIP genes as well as to confirm their tissue-specific expression patterns and inducible expressions under drought and salt stresses. Our results presented here may provide a subset of potential candidate HD-ZIP genes for future engineering modifications of lignocellulosic biomass and stress tolerance characteristics in *Populus*.

## Results and Discussion

### Identification of HD-ZIP gene family in *Populus* and other plant species

To identify putative HD-ZIP genes in *Populus*, we performed a BLASTP search against *Populus* genome release v2.1 using HD-ZIP protein sequences in *Arabidopsis* as queries and the resulting sequences were used as secondary queries. By removing the redundant sequences, 63 HD-ZIP genes were identified in the *Populus* genome. All HD-ZIP candidates were manually analyzed using InterProScan program (http://www.ebi.ac.uk/Tools/InterProScan/) to verify the presence of HD and LZ domain. In a recently published report, a total of 61 HD-ZIP genes were identified in *Populus* by a genome-wide bioinformatics survey [2]. In this study, we further revealed three additional HD-ZIP genes in *Populus* and extended the total member to 63. We designated *Populus* HD-ZIP genes as *PtrHox* following the nomenclature proposed in the previous study [56]. The identified HD-ZIP genes in *Populus* encode proteins ranging from 170 to 855 amino acids (aa) in length with an average of 465 aa. In most cases, there are two or more *Populus* HD-ZIP genes for the orthologues in *Arabidopsis*, but in some cases, there are no orthologous *Populus* HD-ZIP genes in *Arabidopsis*. The detailed information of HD-ZIP family genes in *Populus*, including accession numbers and similarities to their *Arabidopsis* orthologues was listed in Table 1.

**Table 1:** HD-ZIP gene family in *Populus*.

Gene symbol	Gene model (V2.1)	Gene model (V1.1)	*Arabidopsis* orthologue locus	*Arabidopsis* locus description	Score	E-value
PtrHox1	POPTR_0001s02030.1	fgenesh4_pg.C_scaffold_29000247	AT5G46880.1	HB7, HDG5	775	0
PtrHox2	POPTR_0001s11380.1	eugene3.00010699	AT2G46680.1	ATHB-7	131	3E-31
PtrHox3	POPTR_0001s15490.1	eugene3.00290072	AT4G16780.1	ATHB-2, HAT4	335	2E-92
PtrHox4	POPTR_0001s18470.1	fgenesh4_pm.C_LG_I000546	AT1G79840.1	GL2	941	0
PtrHox5/PCN	POPTR_0001s18930.1/HB5	fgenesh4_pm.C_LG_I000560	AT1G52150.1	ATHB-15, CNA	1467	0
PtrHox6	POPTR_0001s23680.1	gw1.148.81.1	AT5G06710.1	HAT14	268	4E-72
PtrHox7	POPTR_0001s38120.1/HB4	estExt_fgenesh4_pg.C_LG_I2905	AT2G34710.1	PHB, ATHB-14	1357	0
PtrHox8	POPTR_0001s40540.1	gw1.146.127.1	AT5G53980.1	ATHB-52	114	2E-26
PtrHox9	POPTR_0001s44540.1	gw1.XIII.2898.1	AT4G04890.1	PDF2	420	e-117
PtrHox10	POPTR_0002s10080.1	estExt_fgenesh4_pm.C_LG_II0461	AT4G40060.1	ATHB-16	191	6E-49
PtrHox11	POPTR_0002s11410.1	estExt_fgenesh4_pg.C_LG_II1036	AT4G37790.1	HAT22	210	8E-55
PtrHox12	POPTR_0002s13720.1	estExt_fgenesh4_pm.C_LG_II0606	AT3G60390.1	HAT3	285	2E-77
PtrHox13	POPTR_0002s15610.1	eugene3.00021438	AT4G00730.1	ANL2, AHDP	1057	0
PtrHox14	POPTR_0002s17680.1	fgenesh4_pm.C_LG_II000817	AT2G46680.1	ATHB-7	219	1E-57
PtrHox15	POPTR_0002s23080.1	estExt_fgenesh4_pm.C_LG_II1004	AT1G05230.2	HDG2	1154	0
PtrHox16	POPTR_0003s04860.1/HB6	estExt_fgenesh4_pg.C_LG_III0436	AT1G52150.1	ATHB-15, CNA	1488	0
PtrHox17	POPTR_0003s05100.1	estExt_fgenesh4_pg.C_LG_III0408	AT1G79840.1	GL2	948	0
PtrHox18	POPTR_0003s07760.1	fgenesh4_pm.C_LG_III000187	AT4G16780.1	ATHB-2, HAT4	320	8E-88
PtrHox19	POPTR_0003s09470.1	gw1.III.2514.1	AT5G46880.1	HB7, HDG5	836	0
PtrHox20	POPTR_0003s14700.1	/	AT2G46680.1	ATHB-7	110	5e-25
PtrHox21	POPTR_0004s01980.1	fgenesh4_pg.C_LG_IV000079	AT4G04890.1	PDF2	1186	0
PtrHox22	POPTR_0004s07310.1	gw1.IV.3966.1	AT1G73360.1	HDG11,EDT1	631	0
PtrHox23/PRE	POPTR_0004s22090.1/HB2	estExt_Genewise1_v1.C_660759	AT5G60690.1	REV, IFL	1375	0
PtrHox24	POPTR_0005s07290.1	/	AT4G40060.1	ATHB-16	244	4E-65
PtrHox25	POPTR_0005s12760.1	estExt_fgenesh4_pg.C_570199	AT2G18550.1	HB2, ATHB-21	202	9E-53
PtrHox26	POPTR_0005s17390.2	estExt_Genewise1_v1.C_LG_V4715	AT4G40060.1	ATHB-16	186	9E-48
PtrHox27	POPTR_0005s19080.1	gw1.V.4364.1	AT4G37790.1	HAT22	265	2E-71
PtrHox28	POPTR_0006s07100.1	fgenesh4_pg.C_LG_VI000532	AT5G06710.1	HAT14	163	8E-41
PtrHox29	POPTR_0006s11890.1	gw1.28.964.1	AT5G03790.1	ATHB51, LMI1	181	4E-46
PtrHox30	POPTR_0006s20820.1	fgenesh4_pm.C_LG_VI000496	AT5G06710.1	HAT14	249	1E-66
PtrHox31	POPTR_0006s25390.1/HB8	estExt_fgenesh4_pm.C_LG_VI0713	AT4G32880.1	ATHB-8	1404	0
PtrHox32	POPTR_0007s01370.1	fgenesh4_pg.C_LG_VII000112	AT4G36740.1	HB-5, ATHB40	78	3E-15
PtrHox33	POPTR_0007s05010.1	gw1.VII.1686.1	AT2G22430.1	ATHB-6	248	4E-66
PtrHox34	POPTR_0007s12450.1	grail3.0019015601	AT4G36740.1	HB-5, ATHB40	208	2E-54
PtrHox35	POPTR_0007s14590.1	estExt_Genewise1_v1.C_LG_VII0041	AT4G37790.1	HAT22	299	1E-81
PtrHox36	POPTR_0008s12840.1	/	AT2G01430.1	ATHB-17	206	1E-53
PtrHox37	POPTR_0008s14770.1	estExt_fgenesh4_pg.C_LG_VIII1313	AT1G69780.1	ATHB-13	333	1E-91
PtrHox38	POPTR_0008s19900.1	eugene3.00081839	AT5G15150.1	ATHB-3, HAT7	122	4E-28
PtrHox39	POPTR_0009s01990.1/HB1	gw1.IX.4748.1	AT5G60690.1	REV, IFL	1367	0
PtrHox40	POPTR_0009s02850.1	/	AT5G06710.1	HAT14	242	3E-64
PtrHox41	POPTR_0010s10340.1	estExt_fgenesh4_pg.C_LG_X0852	AT1G69780.1	ATHB-13	308	2E-84
PtrHox42	POPTR_0010s12300.1	eugene3.00101057	AT2G01430.1	ATHB-17	214	3E-56
PtrHox43	POPTR_0011s00520.1	eugene3.00110213	AT4G04890.1	PDF2	1164	0
PtrHox44	POPTR_0011s10070.1/HB3	estExt_fgenesh4_pg.C_2360002	AT2G34710.1	PHB, ATHB-14	1351	0
PtrHox45	POPTR_0011s11510.1	gw1.XI.3740.1	AT5G53980.1	ATHB-52	114	4E-26
PtrHox46	POPTR_0012s03080.1	gw1.XII.940.1	AT2G46680.1	ATHB-7	147	5E-36
PtrHox47	POPTR_0012s03550.1	estExt_fgenesh4_pm.C_LG_XII0033	AT1G73360.1	HDG11,EDT1	885	0
PtrHox48	POPTR_0012s07270.1	grail3.0015005801	AT3G01470.1	ATHB-1, HAT5	145	4E-35
PtrHox49	POPTR_0012s13390.1	fgenesh4_pg.C_LG_XII001156	AT4G00730.1	ANL2, AHDP	838	0
PtrHox50	POPTR_0014s04460.1	/	AT3G60390.1	HAT3	263	8E-71
PtrHox51	POPTR_0014s07130.1	fgenesh4_pg.C_LG_XIV000202	AT4G00730.1	ANL2, AHDP	1045	0
PtrHox52	POPTR_0014s09860.1	eugene3.00140486	AT2G46680.1	ATHB-7	237	5E-63
PtrHox53	POPTR_0014s15010.1	estExt_fgenesh4_pm.C_LG_XIV0411	AT1G05230.2	HDG2	1174	0
PtrHox54	POPTR_0015s05050.1	fgenesh4_pg.C_scaffold_122000084	AT1G73360.1	HDG11,EDT1	917	0
PtrHox55	POPTR_0015s07640.1	fgenesh4_pm.C_LG_XV000148	AT3G01470.1	ATHB-1, HAT5	135	2E-32
PtrHox56	POPTR_0015s13340.1	eugene3.00151195	AT4G00730.1	ANL2, AHDP	828	0
PtrHox57	POPTR_0016s05890.1	/	AT5G06710.1	HAT14	242	2E-64
PtrHox58	POPTR_0017s01400.1	gw1.44.578.1	AT1G73360.1	HDG11,EDT1	606	e-173
PtrHox59	POPTR_0017s04820.1	eugene3.00640210	AT4G36740.1	HB-5, ATHB40	79	2E-15
PtrHox60	POPTR_0017s11800.1	gw1.XVII.1044.1	AT5G15150.1	ATHB3, HAT7	248	3E-66
PtrHox61	POPTR_0018s08110.1/HB7	fgenesh4_pg.C_LG_XVIII000250	AT4G32880.1	ATHB-8	1389	0
PtrHox62	POPTR_0018s13300.1	eugene3.00181192	AT5G06710.1	HAT14	155	2E-38
PtrHox63	POPTR_0149s00220.1	gw1.XVI.1869.1	AT5G06710.1	HAT14	239	1E-63

Gene loci are from the Phytozome website (http://www.phytozome.net/poplar, release 2.1). A complete list of the coding sequences (CDS), deduced amino acid sequences and genomic DNA sequences is available in Table S1.

In order to gain insights into the evolutionary relationships among plant HD-ZIP proteins, we identified HD-ZIP genes from seven other plant species with whole genome sequences available, including moss (*Physcomitrella patens*), the monocotyledonous angiosperms *Oryza sativa*, *Sorghum bicolor* and *Brachypodium distachyon*, and the dicotyledonous angiosperms *Arabidopsis thaliana*, *Medicago truncatula* and *Vitis vinifera*. Strikingly, HD-ZIP gene family is apparently land plant-specific. All angiosperm genomes as well as the genome of the moss contained genes encoding HD-ZIP proteins, while no representatives were found in algae. A complete list of all HD-ZIP genes identified in the present study was provided in Table S2.

The number of HD-ZIP genes in *Populus* (63) is roughly 1.31 fold than that of *Arabidopsis* (48) and it is only second to its closest woody perennial grape (*Vitis vinifera*), which possesses 65 HD-ZIP genes. This expansion to more abundant HD-ZIP genes in *Populus* and grape genome suggests a great need of HD-ZIP genes to participate in more complicated transcriptional regulations of these two woody species.

### Phylogenetic analysis of HD-ZIP gene family

**Figure 1:**
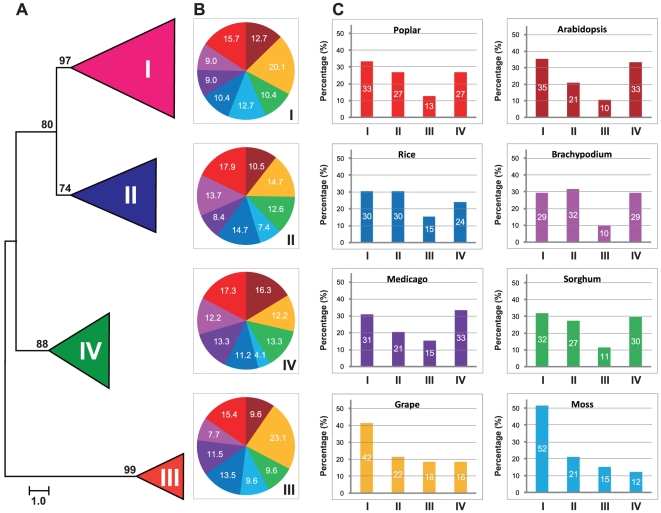
Phylogeny and distribution of HD-ZIP protein from eight plant species. **A.** Phylogenetic tree of HD-ZIP proteins from *Arabidopsis*, rice, *Medicago*, sorghum, *Brachypodium*, *Populus*, *Vitis*, and moss. Phylogeny was constructed by PhyML using maximum likelihood analysis. Bootstrap support values as percentage, are shown on selected major branches. The scale bar indicates the estimated number of amino acid substitutions per site. **B.** Percentage representation of HD-ZIP across the eight plant species within each subfamily. Colors correspond to the plant taxa as listed in C. C: Percentage representation of distributions for HD-ZIP within each plant species.

The abundance of *Populus* HD-ZIP genes to other plant species may derive from multiple gene duplication events, represented by a whole-genome duplication following multiple segmental and tandem duplications [57]. To verify this hypothesis, we first constructed a maximum likelihood phylogenetic tree by PHYML using the full-length HD-ZIP protein sequence alignments of eight different plant species to unveil the evolutionary relationships among plant HD-ZIP proteins. The HD-ZIP proteins of all eight plant species were classified into four well-conserved subfamilies, HD-ZIP I to IV (Fig. 1A), the same as described previously and with significant statistical support [7], [8]. The phylogenetic tree revealed that the plant HD-ZIP sequence distribution predominates with species bias (Fig. 1B). HD-ZIP I genes generally consisted of the largest subfamilies in the plant species except for *Brachypodium* and *Medicago* where HD-ZIP II and IV were the largest respectively. In contrast, HD-ZIP III genes composed of the fewest numbers of HD-ZIP members except for moss. It also appears that the numbers of *Populus* and grape HD-ZIP I and II genes were larger than these of other species. For instance, there were 27 and 21 HD-ZIP I members, 14 and 17 HD-ZIP II members in *Populus* and grape, respectively. In contrast, only 17 and 14 members in HD-ZIP I subfamily, 10 and 14 members in HD-ZIP II subfamily were present in *Arabidopsis* and rice, respectively. Species bias was also evident in subfamily IV. *Populus* and *Arabidopsis* were predominated with 17 and 16 HD-ZIP IV members but grape had only 12 HD-ZIP IV members nonetheless the number of grape HD-ZIP genes was larger than that of other species. In contrast to the much fewer members in subfamily IV, the number of grape HD-ZIPs showed an overwhelming predominance with 12 members present in subfamily III. Similarly, the number of moss HD-ZIPs in subfamily IV was particularly lower with four members compared to that of other species.

**Figure 2:**
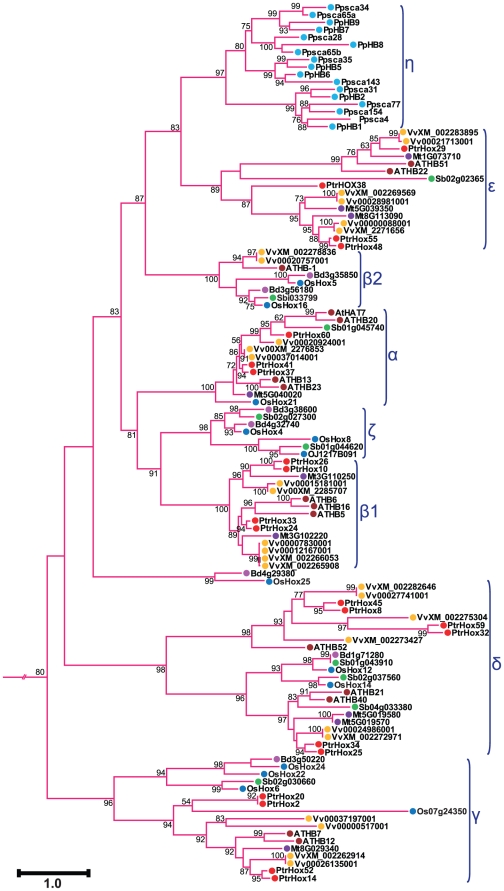
Phylogenetic relationship of HD-ZIP I subfamily from eight plant species. Expanded view of the phylogeny of HD-ZIP I members from Figure 1A. Numbers at each branch indicate bootstrap values and only values higher than 50% are shown. Scale bar corresponds to the estimated number of amino acid substitutions per site. Filled circles represent HD-ZIP proteins from different plant species with colors corresponding to plant taxa as indicated in Figure 1.

**Figure 3:**
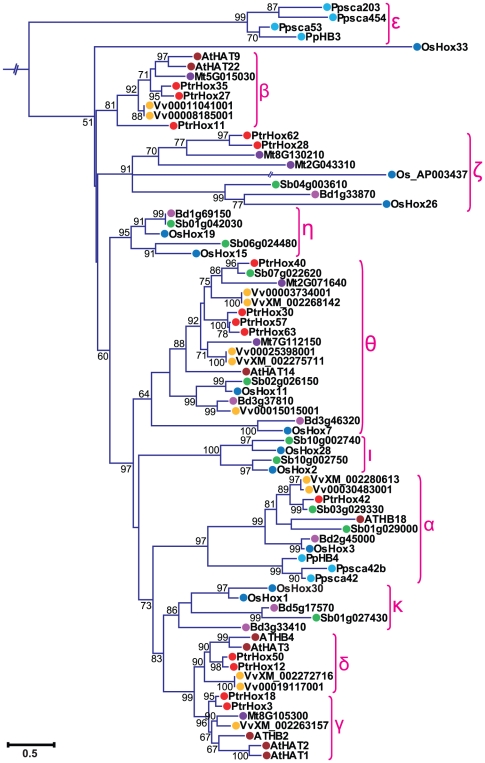
Phylogenetic relationship of HD-ZIP II subfamily from eight plant species. Expanded view of the phylogeny of HD-ZIP II members from Figure 1A. The numbers at the nodes represent the bootstrap values (>50%) from 100 replicates. Scale bar indicates the estimated number of amino acid substitutions per site. Filled circles represent HD-ZIP proteins from different plant species with colors corresponding to plant taxa as indicated in Figure 1.

**Figure 4:**
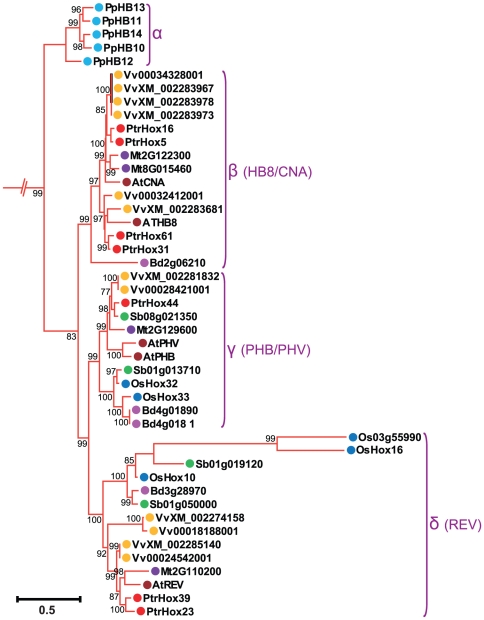
Phylogenetic relationship of HD-ZIP III subfamily from eight plant species. Enlarged view of the phylogeny of HD-ZIP III members from Figure 1A. Numbers at each branch indicate bootstrap values and only values higher than 50% are shown. Scale bar corresponds to the estimated number of amino acid substitutions per site. Filled circles represent HD-ZIP proteins from different plant species. Colors correspond to plant taxa as indicated in Figure 1.

**Figure 5:**
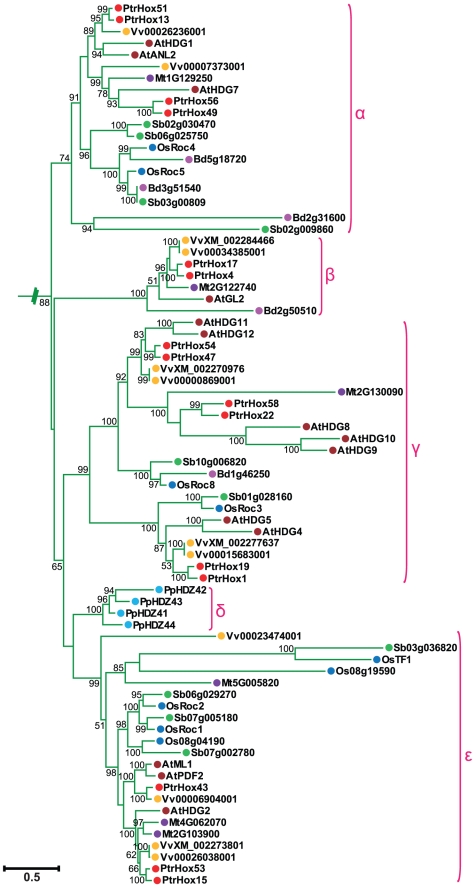
Phylogenetic relationship of HD-ZIP IV subfamily from eight plant species. Enlarged view of the phylogeny of HD-ZIP IV members from Figure 1A. The numbers at the nodes represent the bootstrap values (>50%) from 100 replicates. Scale bar indicates the estimated number of amino acid substitutions per site. Filled circles represent HD-ZIP proteins from different plant species. Colors correspond to plant taxa as indicated in Figure 1.

We further examined the subgroups within each HD-ZIP subfamilies. Consistent with the nomenclature in previous studies of *Arabidopsis* and rice [9], [56], HD-ZIP I subfamily was divided into seven clades designated as clade α, β1, β2, γ, δ, ε, ζ, and η (Fig. 2). Clade η included 17 moss HD-ZIP I members clustered in the most basal clade with high statistical support, whereas clade β1 was exclusively from eudicots (*Arabidopsis thaliana*, *Medicago truncatula*, *Vitis vinifera*, and *Populus*) and clade ζ entirely from monocots (*Oryza sativa*, *Sorghum bicolor*, and *Brachypodium distachyon*). HD-ZIP II subfamily was divided into ten clades, from α through κ (Fig. 3), with clades α to γ designated in our study corresponding to the four major clades of *Arabidopsis* HD-ZIP II proteins as previously analyzed [22]. The ε clade constituted a basal grade exclusively containing the moss members. The other three moss HD-ZIPs clustered together with members from eudicots and monocots in clade α. Several clades including β, γ, and δ were composed of HD-ZIP proteins exclusively from eudicots. Similarly, clades η, ι, and κ were exclusively formed of proteins from monocot species. In contrast, clades θ and ζ included HD-ZIP proteins from both monocot and eudicot species. HD-ZIP III subfamily was classified into four clades designated as α, β, γ, and δ (Fig. 4). This classification generally agrees with the definitions of previous studies [29], [58], [59], [60]. Clade α had a basal node exclusively containing five moss members. Clade β included *Arabidopsis ATHB8* and *ATHB15/CNA* as well their orthologues in the other seven plant species, and clade γ contained *PHB*, *PHV* and their corresponding members. Subgroup δ corresponded to the *REV* clade described in previous studies [29], [58], [60] and can be divided into eudicot- and monocot-specific subclades. HD-ZIP IV subfamily was clustered into four individual subgroups, designated clade α, β, γ, δ, and ε as in a previous study [46] (Fig. 5). Most of the HD-ZIP IV members have been functionally characterized in *Arabidopsis*. Clade α consisted of *Arabidopsis ANL2*, a regulator of anthocyanin accumulation in leaf sub-epidermal layer and of cell identity in root [53]. Clade β included *Arabidopsis GL2* [47], [48], [52] and its orthologues, and Clade γ contained trichome formation genes, *HDG4*, *HDG5*, and *HDG8–12* [46]. Clade ε was composed of *AtML1* and *PDF2* responsible for shoot epidermal cell differentiation [51]. Clade δ, placed between clade β and ε, consisted of four HD-ZIP III genes from moss.

Tree topology displayed that the majority of HD-ZIP genes tend to cluster in eudicot- and monocot-specific patterns, especially within subgroups harboring large members, i.e., clades β2, γ, and ε in HD-ZIP I subfamily, clades γ and δ in HD-ZIP III, and clades α, γ, and ε in HD-ZIP IV. This substantial lineage-specific pattern suggests that HD-ZIP genes in these subgroups may be expanded and then diversified after the monocot-eudicot radiation so that these genes were acquired or differentially retained in eudicot genomes after their divergences from monocots. Nonetheless, a small number of the HD-ZIP members were presented in both monocots and eudicots in several clades such as clades θ and ζ in HD-ZIP II, which suggests that the expansion of these HD-ZIPs might predate the divergence of eudicots and monocots.

Phylogenetic tree topology further revealed that 28 *Populus* HD-ZIP pairs at the terminal nodes of each subfamily shared high degrees of sequence similarities and were assigned as paralogous pairs (homologous genes that diverged by gene duplication) (Fig. 2, 3, 4, and 5). These 28 paralogous pairs of HD-ZIP proteins, accounting for more than 89% of the entire HD-ZIP family, had sequence similarity ranging from 62% to 95% (Table S3).

### Chromosomal location and gene duplication of *Populus* HD-ZIP genes

**Figure 6:**
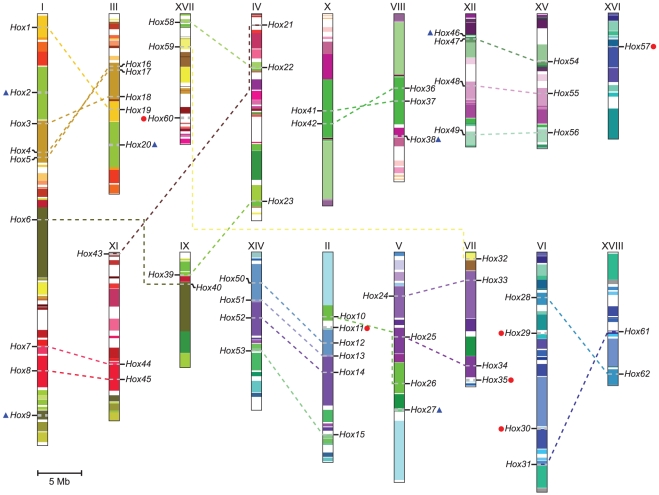
Chromosomal locations and segmental duplication events of *Populus* HD-ZIP genes. The schematic diagram of genome-wide chromosome organization arisen from the salicoid genome duplication event in *Populus* was accomplished based on duplication coordinates from the *Populus* genome assembly v2.1. Segmental duplicated blocks are indicated with the same colors. The duplicated paralogous pairs of HD-ZIP are connected with dotted lines. Blue triangles indicate HD-ZIPs located on duplicated segments with the corresponding member lost. Red circles represent HD-ZIPs located out of any duplicated regions. Scale represents a 5 Mb chromosomal distance.

In silico mapping of the gene loci showed that totally 62 *Populus* HD-ZIP genes were mapped to linkage groups (LG) currently, with only one gene (*PtrHox63*) remained on as yet unmapped scaffolds (Fig. 6). The 62 *Populus* HD-ZIP genes were distributed across all LGs, except for LGXIII and IXI. LG I had the largest number of nine HD-ZIP genes followed by six on LG II and five on LG III, respectively. In contrast, only one HD-ZIP gene was found on LG XVI and two HD-ZIP genes on LG IX, XI, and XVIII, respectively. No substantial clustering of *Populus* HD-ZIP genes was present, even on the LGs with high densities of HD-ZIP genes.

Previous studies revealed that *Populus* genome has undergone at least three rounds of genome-wide duplications followed by multiple segmental duplication, tandem duplication, and transposition events such as retroposition and replicative transposition [57]. Particularly, the segmental duplication associated with the salicoid duplication event occurred 65 million years (MY) ago remarkably contributed to the expansion of many multi-gene families [61], [62], [63], [64], [65], [66]. To determine the possible relationship between the HD-ZIP genes and potential segmental duplications, we mapped *Populus* HD-ZIPs to the duplicated blocks established in the previous studies [57]. The distributions of HD-ZIP genes relative to the corresponding duplicate blocks were illustrated in Figure 6. Within the identified duplicated blocks associated with the recent salicoid duplication event, about 81% (50 of 62) of *Populus* HD-ZIPs were preferentially retained duplicates that located in both duplicated regions. Six duplicated blocks only contained HD-ZIPs (*PtrHox2*, *9*, *20*, *27*, and *38*) on one of the blocks and lacked duplicates on the corresponding block, suggesting that dynamic rearrangement may have occurred following the segmental duplication, which led to loss of some of the genes. In contrast, only a small number of six HD-ZIP genes (*PtrHox11*, *29*, *30*, *35*, *57*, and *60*) were located outside of any duplicated blocks. None of HD-ZIP genes was represented in distinct tandem duplicate gene clusters, indicating that tandem duplications do not seem to play an important role on the expansion of the HD-ZIP gene family in *Populus*.

Evidence of salicoid segmental duplications was present in all HD-ZIP subfamilies, particularly in the HD-ZIP III and HD-ZIP IV subfamilies. HD-ZIP III subfamily had the highest rate of segmental duplications among the four subfamilies, with every member impacted by segmental duplications. Similarly, the vast majority genes in HD-ZIP IV except for *PtrHox9*, accounting for 94.7% of the total subfamily, were impacted by segmental duplications. In comparison, segmental duplications were relatively under-presented in subfamilies HD-ZIP I and II, with rates of 70% (14 out of 20) and 67% (12 out of 18), respectively.

To explore whether other mechanisms contributed towards the expansion of the duplicated genes, we also searched for the presence of transposons and retrotransposons in the flanking genomic sequences of 10-kb upstream and downstream of each HD-ZIP gene. However, there was very limited contribution of transposons/retrotransposons to the expansion of HD-ZIP gene family in *Populus* (data not shown).

Based primarily on the genomic organization of HD-ZIP genes, we are attempting to speculate that segmental duplications exclusively contributed to the expansion of *Populus* HD-ZIP gene family. Similarly, segmental duplications have also been shown to contribute to the expansion of other multi-gene families in *Populus* [61], [62], [63], [64], [65], [66], [67]. Our results indicated that *Populus* HD-ZIP genes have been preferentially retained at a relatively high rate of 81%. This number is much higher than the average rate following the salicoid genome-wide duplication in the *Populus* lineage, in which approximately 33% of predicted genes are retained in duplications resulting from the salicoid duplication event on the genome-wide scale [57]. The high retention rates of duplicated genes are also found in other gene families of *Populus* [62], [63], [68]. These findings corroborates previous findings that genes involved in transcription regulation and signal transduction are preferentially retained following duplications [69], [70], [71]. Another plausible explanation to the relatively high retention rate of duplicate genes in HD-ZIP gene family may lie in the fact that *Populus* genome has been indicated to evolve at a much slower rate compared to *Arabidopsis* [57].

As a large proportion of HD-ZIP proteins appear to be paralogous pairs unveiled by the phylogenetic analysis, we further investigated whether traceable genome duplication events have contributed to the expansion of the HD-ZIP family. Of 28 HD-ZIP paralogous pairs we examined, 25 paralogous pairs remained in conserved positions on segmental duplicated blocks (Fig. 6), suggesting that these 25 paralogous pairs may be derived from segmental duplication event during the evolutionary process. No traceable duplication events could be inferred for only three paralogous pairs (*PtrHox2/20*, *PtrHox27/35*, and *PtrHox30/57*). Among them, three genes (*PtrHox30*, *35*, and *57*) were located outside of any segmental duplication blocks. Although the other three genes (*PtrHox2*, *20*, and *27*) were located on the duplicated blocks, their paralogous counterparts appeared to have lost from the *Populus* genome.

Duplicated genes may undergo divergent fates such as nonfunctionalization (loss of original functions), neofunctionalization (acquisition of novel functions), or subfunctionalization (partition of original functions) during subsequent evolution [72], [73]. To explore whether Darwinian positive selection was involved in HD-ZIP gene divergence after duplication, the substitution rate ratios of nonsynonymous (dN or Ka) versus synonymous (dS or Ks) mutations (dN/dS or Ka/Ks) were calculated for 28 paralogous pairs. Generally, Ka/Ks = 1 means that the genes are pseudogenes with neutral selection, Ka/Ks<1 indicates the functional constraint with negative or purifying selection of the genes, and Ka/Ks>1 shows the accelerated evolution with positive selection. In this study, the Ka/Ks ratios from 24 segmental duplication pairs were less than 0.4 (Table 2). Only one duplication pair *PtrHox32/59* showed the Ka/Ks ratio larger than 0.4 (Table 2). Another paralogous gene pair *PtrHox2/20*, which were located out of segmental duplication blocks established in current *Populus* genome assembly (v2.1), had the Ka/Ks ratio slightly larger than 0.5. The relatively higher Ka/Ks ratio of *PtrHox2/20* suggests that they may have experienced relatively rapid evolution following duplication. Based on the Ka/Ks analyses, we could conclude that the *Populus* HD-ZIP gene family have mainly experienced strong purifying selection pressure with limited functional divergence occurred after segmental duplications. However, it still remains unknown whether the duplicated HD-ZIP genes correspond to genetic redundancy or have evolved divergent functions. Based on the divergence rate of 9.1×10^−9^ synonymous mutations per synonymous site per year as previously proposed for *Populus* [74], duplications of these 28 paralogous pairs was estimated to have occurred between 9.75 to 24.96 million year (MY) ago (Table 2).

**Table 2:** The Ka/Ks ratios and estimated divergence time for paralogous HD-ZIP proteins.

Paralogous pairs	Ka	Ks	Ka/Ks	Duplication date (MY)
PtrHox1–PtrHox19	0.054	0.217	0.248	11.91
PtrHox10–PtrHox26	0.074	0.314	0.235	17.23
PtrHox12–PtrHox50	0.046	0.207	0.221	11.39
PtrHox13–PtrHox51	0.027	0.275	0.099	15.12
PtrHox14–PtrHox52	0.049	0.308	0.158	16.91
PtrHox15–PtrHox53	0.029	0.284	0.102	15.62
PtrHox2–PtrHox20	0.196	0.360	0.543	19.79
PtrHox21–PtrHox43	0.033	0.244	0.135	13.39
PtrHox22–PtrHox58	0.116	0.296	0.393	16.27
PtrHox23–PtrHox39	0.047	0.212	0.219	11.64
PtrHox24–PtrHox33	0.042	0.278	0.150	15.25
PtrHox25–PtrHox34	0.083	0.219	0.379	12.03
PtrHox27–PtrHox35	0.122	0.399	0.306	21.90
PtrHox28–PtrHox62	0.144	0.454	0.316	24.96
PtrHox3–PtrHox18	0.040	0.367	0.110	20.15
PtrHox30–PtrHox57	0.060	0.314	0.190	17.23
PtrHox31–PtrHox61	0.020	0.219	0.092	12.04
PtrHox32–PtrHox59	0.097	0.197	0.492	10.83
PtrHox36–PtrHox42	0.054	0.198	0.271	10.85
PtrHox37–PtrHox41	0.050	0.313	0.158	17.20
PtrHox4–PtrHox17	0.040	0.191	0.208	10.50
PtrHox47–PtrHox54	0.031	0.223	0.141	12.27
PtrHox48–PtrHox55	0.042	0.342	0.122	18.78
PtrHox49–PtrHox56	0.063	0.178	0.357	9.75
PtrHox5–PtrHox16	0.017	0.224	0.076	12.32
PtrHox6–PtrHox40	0.081	0.273	0.296	15.01
PtrHox7–PtrHox44	0.025	0.190	0.131	10.43
PtrHox8–PtrHox45	0.119	0.369	0.322	20.27

### Gene structure and conserved motifs of *Populus* HD-ZIP genes

**Figure 7:**
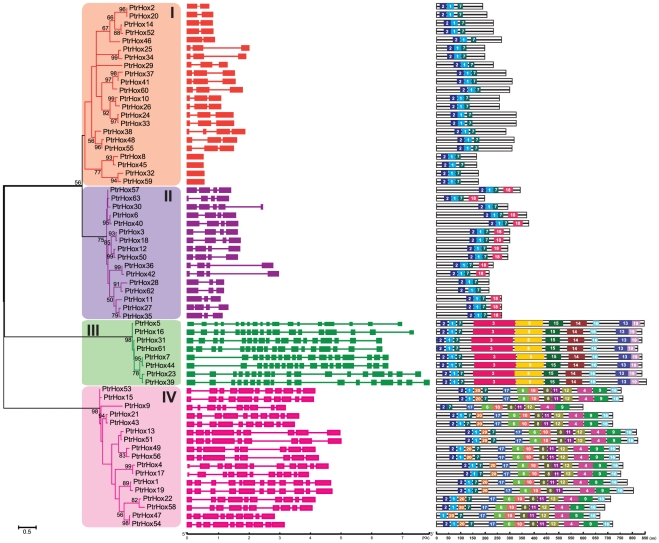
Phylogenetic relationship, gene structure and motif compositions of *Populus* HD-ZIP genes. **A.** The phylogenetic tree was constructed using full-length protein sequences by the maximum likelihood method with 100 bootstrap replicates. The percentage bootstrap scores higher than 50% are indicated on the nodes. The four major phylogenetic subfamilies designated as I to IV are marked with different color backgrounds. **B.** Exon/intron structures of HD-ZIP genes from *Populus*. Exons and introns are represented by green boxes and black lines, respectively. The sizes of exons and introns are proportional to their sequence lengths. **C.** Schematic representation of the conserved motifs in the HD-ZIP proteins from *Populus* elucidated by MEME. Each motif is represented by a number in the colored box. The details of individual motif are provided in Table S4.

To gain further insights into the structural diversity of *Populus* HD-ZIP genes, we first constructed a separate phylogenetic tree exclusively using the full-length HD-ZIP protein sequences of *Populus*. *Populus* HD-ZIP proteins were also classified into four independent subfamilies as described above (Fig. 7A and Fig. 1A). We then compared the exon/intron organization in the coding sequences of each *Populus* HD-ZIP genes (Fig. 7B). Most closely related *Populus* HD-ZIP members within the same subfamilies shared very similar gene structure in terms of either intron numbers or exon lengths (Fig. 7B), i.e, all *Populus* HD-ZIP I gene had two or three introns, and HD-ZIP III possessed similar number of introns (as many as 17) in their coding sequences. Nonetheless, the gene structures in *Populus* HD-ZIP subfamilies II and IV appeared to be more variable and displayed the largest number of exon/intron structure variants, i.e., four *Populus* HD-ZIP II members had no introns in their coding regions but the other HD-ZIP II possessed one to three introns, and HD-ZIP IV members had a large variation from 7 to 11. We also investigated intron phases with respect to codons. Although the intron phases were remarkably well-conserved within the same subfamilies, there were striking distinctions in the arrangement of introns and intron phases among subfamilies of *Populus* HD-ZIP I–IV (Fig. S1). The conservation of intron phases within *Populus* HD-ZIP subfamilies and the striking dissimilarity between subfamilies may reciprocally lend supports to the results from phylogenetic analysis and genome duplication.

We further examined the exon/intron organization of 25 paralogous pairs in *Populus* HD-ZIP genes to inquire the information of traceable intron gain or loss within these genes. Although 22 paralogous pairs showed conserved exon/intron structures in either intron numbers or gene lengths, three paralogous pairs (*PtrHox27*/*35*, *30*/*57*, and *47*/*54*) exhibited certain degrees of variations (Fig. 7B). These differences might be derived from the single intron loss or gain event during the structural evolution of HD-ZIP paralogues.

To further reveal the diversification of *Populus* HD-ZIP genes, we predicted the conserved motifs using MEME motif detection software and 20 distinct motifs were identified (Table S4). The details of the 20 putative motifs were referred in Table S4. Most of the closely related members in the phylogenetic tree shared common motif compositions with each other, suggesting functional similarities among the HD-ZIP proteins within the same subfamily (Fig. 7C). However, the biological significance of most of the putative motifs remains to be elucidated as they do not have homologues when searching against Pfam (http://pfam.sanger.ac.uk/search) and SMART (Simple Modular Architecture Research Tool) databases (http://smart.embl-heidelberg.de). As illustrated in the previous studies, most of the HD-ZIP proteins possessed HD and LZ domains at the N termini. In this study, motif 1 and 2 specifying HD helices and motif 7 corresponding to the ZIP subdomain were present in all of the HD-ZIP family members in *Populus*. Motif 3 corresponding to the START domain was present in the HD-ZIP III subfamily proteins. The CPSCE motif (motif 18) was found in the majority members in the HD-ZIP II subfamily with the exception of PtrHox28, 57, and 62. The conserved motifs 13 and 19 representing MEKHLA were found to be distributed in the C termini of HD-ZIP III proteins. Members in HD-ZIP I and II possessed significantly reduced number (3–4) of conserved motifs compared to those in HD-ZIP III and IV subfamily (8–10). Particularly, only three motifs (1, 2, and 7) were present in HD-ZIP I proteins, suggesting of high sequence divergence in the other regions of the proteins.

### Differential expression profile of *Populus* HD-ZIP genes

**Figure 8:**
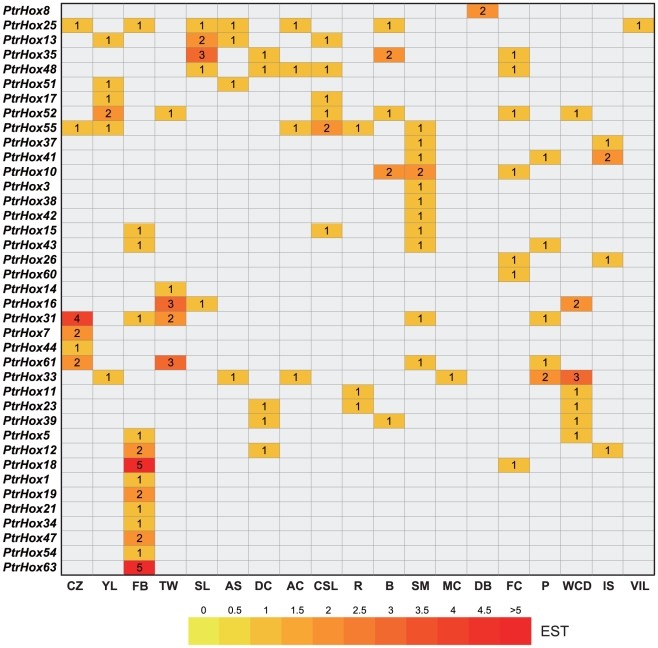
In sillico EST analysis of *Populus* HD-ZIP genes. EST frequency for each gene was calculated by evaluating its EST representation among 19 cDNA libraries available at PopGenIE (http://www.popgenie.org/) [75]. The heatmap was visualized using Heatmapper Plus tool by counting the corresponding ESTs for particular gene in the database. Color bar at bottom represents the frequencies of EST counts. CZ: cambial zone, YL: young leaves, FB: flower buds, TW: tension wood, SL: senescing leaves, AS: apical shoot, DC: dormant cambium, AC: active cambium, CSL: cold stressed leaves, R: roots, B: bark, SM: shoot meristem, MC: male catkins, DB: dormant buds, FC: female catkins, P: petioles, WCD: wood cell death, IS: imbibed seeds, VIS: Virus/fungus-infected leaves.

Publicly available Expressed Sequence Tags (ESTs) provide a useful tool to survey gene expression profiles by the means of Digital Northern. We first carried out a preliminary analysis of HD-ZIP gene expression under various growth conditions and across different tissues by counting the frequencies of ESTs in different *Populus* cDNA libraries (Fig. 8). Completely searching of the digital expression profiles from PopGenIE (http://www.popgenie.org/) [75] yield a total of 39 *Populus* HD-ZIP genes in the cDNA libraries. Not surprisingly and consistent with the usual transcriptional low-abundances of transcription factors [61], the frequencies of these ESTs were relatively low, and most of the HD-ZIPs were represented by only one single EST in the cDNA libraries. Nevertheless, these expression profiles demonstrated that most of the HD-ZIPs have a broad expression pattern across different tissues.

**Figure 9:**
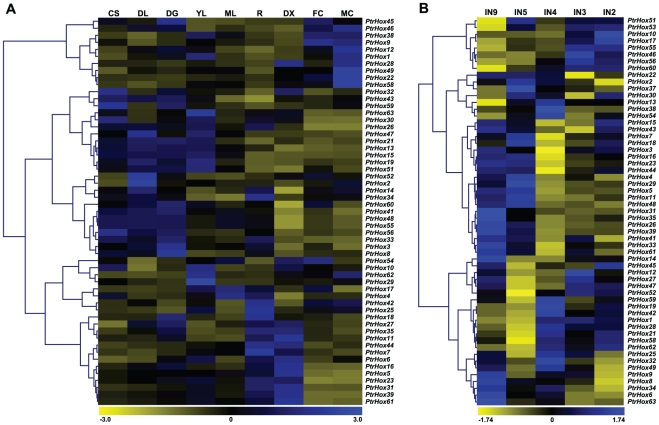
Expression profiles of *Populus* HD-ZIP genes across different tissues. Background corrected expression intensities were log-transformed and visualized as heatmaps (see Materials and Methods). **A.** Heatmap showing hierarchical clustering of 54 *PtrHox* genes across various tissues analyzed. The Affymetrix microarray data were obtained from NCBI Gene Expression Omnibus (GEO) database under the series accession number GSE13990. CL, continuous light-grown seedling; DL, etiolated dark-grown seedling transferred to light for 3 h; DS, dark-grown seedlings; YL, young leaf; ML, mature leaf; R, root; DX, differentiating xylem; FC, female catkins; MC, male catkins. **B.** Heatmap showing hierarchical clustering of 57 *PtrHox* genes at different stem development/growth stages. The NimbleGen microarray data were obtained from NCBI GEO database under the series accession number GSE17230. IN2-IN9, stem internodes 2 to stem internodes 9. Color scale represents log2 expression values, yellow represents low level and blue indicates high level of transcript abundances.

To gain more insights into the expression profiles of HD-ZIP genes, we then re-analyzed the previously published microarray data in *Populus*. We first investigated the global expression profiles of HD-ZIP genes by examining an Affymetrix (GSE13990) [61] and a Nimblegen (GSE13043) [76] microarray data from Gene Expression Omnibus [77]. Although these two microarray dataset were performed in different platforms, they largely represented the *Populus* HD-ZIP genes presenting in this study. Fifty-four HD-ZIP genes were included on both GSE13990 and GSE13043, and only three additional genes (*PtrHox37*, *53*, and *55*) were solely present on GSE13043 (Table S5). The majority of HD-ZIP genes showed a distinct tissue-specific expression pattern (Fig. 9). Of these 57 HD-ZIPs examined, ten genes presented in both microarray datasets had high transcript accumulation in the differentiating xylems and basal stems (internode 9) undergoing secondary growth. Phylogenetic analysis further showed that eight genes (*PtrHox5*, *7*, *16*, *23*, *31*, *39*, *44*, and *61*) in this subset fell into HD-ZIP III subfamily and the other two genes (*PtrHox11* and *35*) into HD-ZIP II (Fig. 3). In *Arabidopsis* and rice, HD-Zip III genes have been functionally well-characterized as developmental regulators of the apical embryo patterning, shoot meristem formation and vascular differentiation [29], [59]. Among them, two closely related members, *ATHB8* and *CNA/ATHB15/ICU4*, have been functionally characterized to regulate vascular patterning and procambial development [30], [32], [36]. In comparison, the eight *Populus* genes from HD-ZIP III subfamily identified in the present study showed preferentially high expression levels in secondary xylem, indicative of their putative roles in the regulation of secondary growth in *Populus*. Recently, two orthologous genes of *REV* (*PRE/PtrHox23*) and *CNA/ATHB15/ICU4* (*PCN/PtrHox5*) were functionally characterized in *Populus* respectively, with specific roles in the secondary cell wall formation and cell fate determination [54], [55]. However, the functional roles of the rest HD-ZIP III genes in *Populus* remain to be elucidated. Besides the large proportion of HD-ZIP genes were highly transcribed in the secondary growth tissues, another 11 genes comprising of four HD-ZIP I genes (*PtrHox46*, *47*, *55*, and *60*), three HD-ZIP II genes (*PtrHox28*, *42*, and *62*) and four HD-ZIP IV genes (*PtrHox1*, *19*, *53*, and *56*) showed comparatively higher transcript abundances in tissues undergoing primary growth in the upper stem (internode 2 to internode 4) (Fig. 9B). How these *Populus* HD-ZIPs perform their functional roles in the primary growth and secondary cell wall formation remains to be elucidated and further functional analyses will be required to understand their biological roles in *Populus*.

*Populus* HD-ZIP may also involve in other biological processes, such as male and female catkin differentiation, root specification and photosynthetic response. Seventeen HD-ZIP genes were preferentially expressed in male and female catkins (Fig. 9A), of which, 11 genes had the highest transcript abundances in male catkins (MC), and six genes in female catkins (FC). Another subset of 11 HD-ZIP genes, comprising of five HD-ZIP I (*PtrHox8*, *14*, *26*, *33*, and *34*), four HD-ZIP II (*PtrHox11*, *18*, *27*, and *35*), and two HD-ZIP III genes (*PtrHox7* and *44*) displayed biased expression in root tissue. In addition, four HD-ZIP genes (*PtrHox2*, *18*, *45*, and *47*) were differentially expressed in dark-grown etiolated seedlings (DG) and continuous light grown seedlings (CL), suggesting their putative roles in photoperiodic regulation.

**Figure 10:**
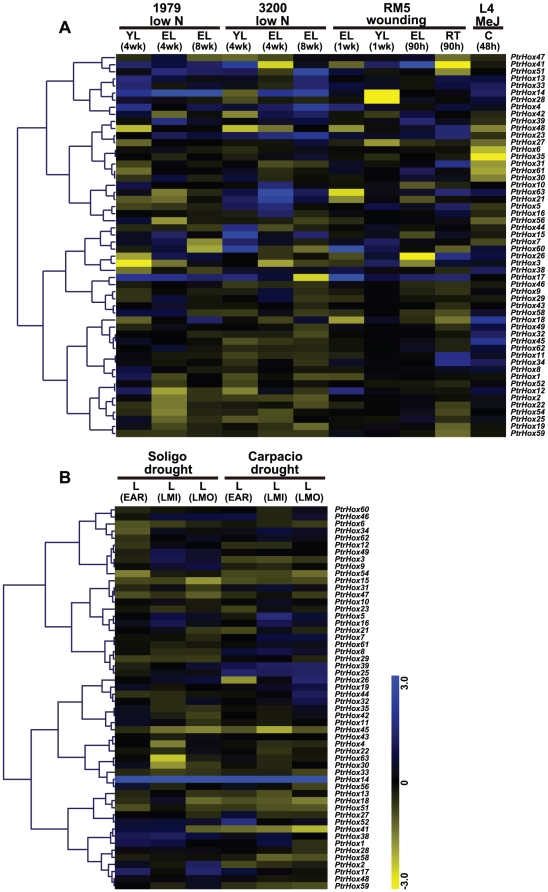
Differential expression of *Populus* HD-ZIP genes under different abiotic stresses. Expression is indicated as fold-change of experimental treatments relative to control samples and visualized in heatmaps (see Materials and Methods). Color scale represents log2 expression values, yellow represents low level and blue indicates high level of transcript abundances. **A.** Heatmap showing hierarchical clustering of 54 *PtrHox* genes across various tissues and genotypes analyzed. Microarray data under the series accession number GSE16786 was obtained from NCBI GEO database. Genotypes analyzed included: *P. fremontii*×*angustifolia* clones 1979, 3200, and RM5, *P. tremuloides* clones 271 and L4, and *Populus deltoids* clones Soligo and Carpaccio. Tissues analyzed included: YL, young leaves; EL, expanding leaves; ML, mature leaves; RT, root tips; C, suspension cell cultures. Stress treatments included: low N, nitrogen limitation; MeJ, Methyl Jasmonate elicitation; wounding, sampled either one week or 90 hours after wounding. **B.** Heatmap showing hierarchical clustering of 54 *PtrHox* genes under short-term and long-term water deficit. Microarray data under the series accession number GSE17230 was obtained from NCBI GEO database. EAR, early response (EAR) to water deficit by 36 hours; LMI, long-term (10-day) response to mild stress with soil relative extractable water (REW) at 20–35%; LMO, long-term (10-day) response to moderate stress with soil relative extractable water (REW) at 10–20%.

To further investigate the responses of *Populus* HD-ZIP genes to abiotic stresses, we also examined their expression patterns under abiotic stresses including low nitrogen, mechanical wounding, drought, as well as Methyl Jasmonate (MeJ) treatment. Gene expression of most HD-ZIP genes were induced or suppressed under these abiotic stresses (Fig. 10). *PtrHox14* in HD-ZIP I subfamily was commonly up-regulated under both nitrogen deprivation and drought stress treatments in two different *Populus* genotypes. However, four genes (*PtrHox11*, *22*, *54*, and *59*) were down-regulated under nitrogen deprivation stress with four-week old young leaves (YL), with four-week and eight-week old expanded leaves (EL) in both genotypes 1979 and 3200 (Fig. 10A).

The responses of HD-ZIP genes to nitrogen deficit stress differ between the two *Populus* genotypes examined. For instance, three genes namely *PtrHox1*, *PtrHox8* and *PtrHox12* were significantly up-regulated at four-week old leaves in genotype 1979, whereas no distinctive expression patterns were observed in genotype 3200 (Fig. 10A). Mechanical wounding stress caused commonly up-regulation of two genes (*PtrHox17* and *23*) and down-regulation of *PtrHox27* at 90 h and/or one week after wounding in young leaves, expanding leaves and root tips. In addition, a subset of genes showed up- or down-regulation only under mechanical wounding stress with young leaves. Similarly, transcripts of a considerable proportion of genes were either enhanced or repressed following mechanical wounding in expanded leaves or root tips, respectively (Fig. 10A). In response to MeJ feeding in cell culture, two genes (*PtrHox18* and *45*) were shown to be significantly up-regulated, whereas the transcripts of seven genes (*PtrHox6*, *30*, *31*, *35*, *48*, *56*, and *61*) showed down-regulation (Fig. 10A). In responses to drought stress, eight genes including three genes from HD-ZIP I (*PtrHox33*, *45*, and *54*), one gene from HD-ZIP II (*PtrHox6*) as well as four genes in HD-ZIP IV subfamilies (*PtrHox15*, *47*, *51*, and *58*) were shown to be down-regulated in both genotypes under all drought conditions tested, which included an early response (EAR) to water deficit by 36 hours and long-term (10-day) response to mild stress (LMI) and moderate stress (LMO) (Fig. 10B).

The high proportion of segmental duplication of HD-ZIP genes and the preferential retention of duplicates raises the question about their functional redundancy. Duplicate genes may have different evolutionary fates: nonfunctionalization, neofunctionalization, or subfunctionalization, which may be indicated with divergence in their expression patterns. Of the 28 paralogous pairs of HD-ZIP genes, five genes do not have corresponding probe sets in the microarray datasets and thus were excluded in further analysis. Twenty-one pairs out of the remaining 23 paralogous pairs were located onto duplicated blocks. Five paralogous pairs (*PtrHox5/16*, *PtrHox7/44*, *PtrHox13/51*, *PtrHox23/39*, and *PtrHox31/61*) derived from segmental duplications shared almost identical expression patterns with respect to different tissues and various stresses. In contrast, the expression patterns of three paralogous pairs (*PtrHox8/45*, *PtrHox21/43*, and *PtrHox22/58*) diversified significantly, indicating substantial neofunctionalization during subsequent evolution processes. For instance, *PtrHox8* gene showed the highest transcript abundances in dark-grown etiolated seedlings and roots, and the least expressions in male and female catkins, whereas its duplicated counterpart *PtrHox45* was preferentially expressed in female catkins and dark-grown etiolated seedlings. Although the expression patterns of the rest of duplicate genes were partially redundant, distinct pattern shifts can be discerned with respect to the microarray datasets investigated, suggests that they might have undergone subfunctionalization. These findings indicated that expression profiles of HD-ZIPs have diverged substantially after segmental duplications, thus we are attempting to speculate that the HD-ZIP genes in *Populus* are likewise to have been retained by substantial subfunctionalization during the evolutionary processes.

### Examination of HD-ZIP gene expressions by qRT-PCR

**Figure 11:**
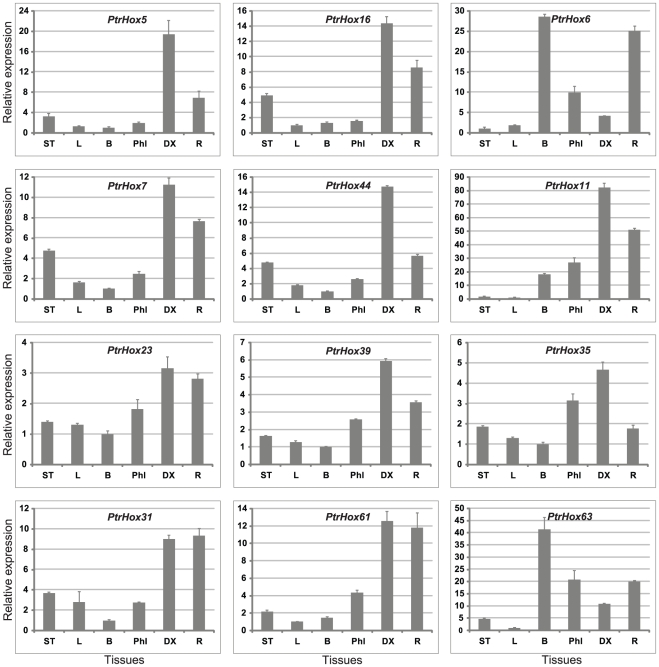
Expression analysis of 12 selected HD-ZIP genes using qRT-PCR. The relative mRNA abundance of 12 selected HD-ZIP genes was normalized with respect to two reference genes *UBQ10* and *UKN1* (*Populus* orthologue of *Arabidopsis AT4G33380*) in six different tissues. Bars represent standard deviations (SD) of three technical replicates. ST, shoot tips; L, leaves from 4–6 stem internodes; Phl, phloem; DX, differentiating xylem; R, roots; B, bark.

To verify the expression profiles of *Populus* HD-ZIP genes obtained by the microarray analysis, qRT-PCR analysis was performed on six different tissues for 12 selected HD-ZIP genes, including four paralogous pairs (*PtrHox5/16*, *PtrHox7/44*, *PtrHox23/39*, and *PtrHox31/61*) that were presumably highly expressed in developing xylem based on microarray analysis. Sequence-specific primers were used to distinguish the amplicons of the paralogous pairs. The gene expression pattern detected by qRT-PCR was roughly in consistency with the microarray analysis and had very distinct tissue-specific expression pattern (Fig. 11). A subset of ten genes were highly expressed in differentiating xylem and weakly expressed in cortex and leaves. Among them, *PtrHox5* (*PCN*) and *PtrHox23* (*PRE*) have been recently functionally characterized [54], [55].

Paralogous pairs with divergent expression patterns could represent the events of sub- or neo-functionalization that may lead to evolutionary diversifications of gene functionalities. To test this hypothesis, we first investigated the divergence of gene expression in the duplicated *PtrHox* gene pairs to reveal the role of segmental duplications. Four paralogous gene pairs we examined showed similar tissue-specific expression patterns. *PtrHox5/16* and *PtrHox7/44* were most abundantly expressed in differentiating xylems, followed by roots and shoot tips, and were least expressed in cortex and leaves tissues. *PtrHox31*/*PtrHox61* genes showed the highest transcript abundances in both differentiating xylems and roots with much lower expressions in cortex and leaf tissues. *PtrHox23*/*PtrHox39* genes were expressed much higher in differentiating xylems and roots than the other tissues. Meanwhile, Ka/Ks ratios of these four paralogous pairs were relatively low with ratios 0.076 for *PtrHox5/16*, 0.092 for *PtrHox31/61*, 0.131 for *PtrHox7/44* and 0.219 for *PtrHox23/39* (Table 2). This tissue-specific expression pattern, combined with the Ka/Ks analysis (Table 2), suggests that the functions of *Populus* HD-ZIP III genes may have been retained with a relatively low divergence.

**Figure 12:**
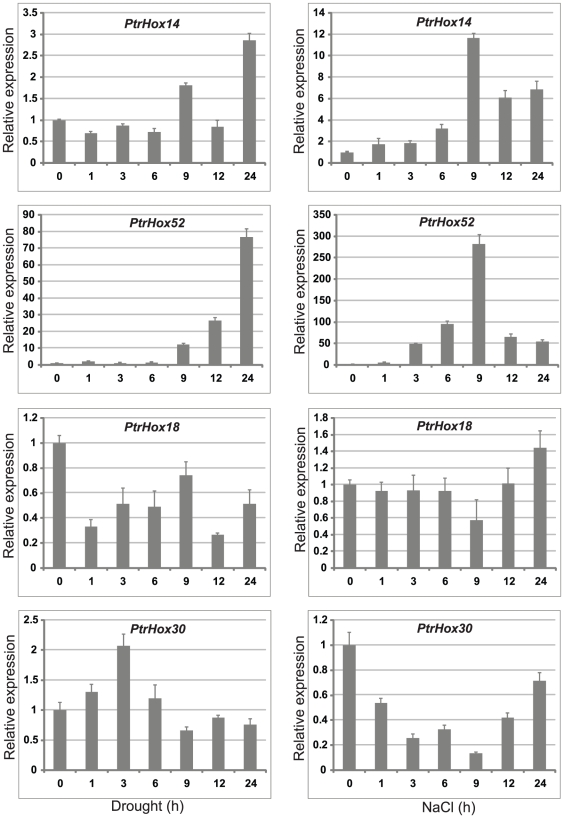
Expression analysis of four selected HD-ZIP genes under drought and salinity stresses using qRT-PCR. The relative mRNA abundance of four selected HD-ZIP genes was normalized with respect to two reference genes *UBQ10* and *PP2a* in drought and salinity stress treatments. Bars represent standard deviations (SD) of three technical replicates. X-axis is time courses of stress treatments for each gene.

Besides the tissue-specific gene expression pattern, the response of four HD-ZIP genes to drought and salinity stresses was also analyzed by qRT-PCR and the results were broadly consistent with those of the microarray data (Fig. 12 and Fig. 10). Response of *PtrHox18* gene was significantly decreased in leaves at different time-points following dehydration stress, while no obvious differential expressions were observed in a 24-hour time-course following salinity stress treatment. *PtrHox30* gene was significantly induced in the first three hours following dehydration stress and then decreased gradually at four time points thereafter, whereas the expression of *PtrHox30* decreased significantly following NaCl treatment with the lowest expression at nine hours after treatment. Similar to microarray analysis, the transcript abundances of paralogous pair *PtrHox14/52*, orthologous to *Arabidopsis ATHB7* involving in ABA-related and abiotic stress responses [13], [15], [16], were both significantly induced in *Populus* leaves 24 hours after dehydration stress and both showed the highest accumulation in leaves nine hours after the treatment of high salinity stress.

Taken all of the evidences together, the expression patterns of *Populus* HD-ZIP genes detected by qRT-PCR are generally consistent with microarray analyses nonetheless two different *Populus* genotypes (*Populus deltoids* and *Populus×euramericana cv*) were used.

## Materials and Methods

### Ethics Statement

No specific permits were required for the described field studies. No specific permissions were required for these locations and activities. The location is not privately-owned or protected in any way and the field studies did not involve endangered or protected species.

### Database search and sequence retrieval

Sequences of *Arabidopsis* HD-ZIP proteins were obtained from the *Arabidopsis* Information Resource (TAIR, http://www.arabidopsis.org/, release 10.0). Rice HD-ZIP gene sequences were downloaded from rice genome annotation database (http://rice.plantbiology.msu.edu/, release 5.0). Sequences of *Populus*, *Medicago*, *Sorghum*, *Brachypodium*, *Vitis*, and *Physcomitrella patens* were downloaded from Phytozome (http://www.phytozome.net/). Local blast was performed using *Arabidopsis* HD-ZIP protein as queries for the identification of the HD-ZIP genes from *Populus* and seven other plant species. For the misannotated genes, manual reannotation was performed using online web server FGENESH (http://linux1.softberry.com/berry.phtml). Then, all the sequences were further manually analyzed to confirm the presence of HD and LZ domain using InterProScan program (http://www.ebi.ac.uk/Tools/InterProScan/).

### Phylogenetic analysis

Multiple sequence alignments of the full-length protein sequences were performed by MAFFT (v6.843b) program. The maximum likelihood (ML) phylogenetic tree was constructed using PhyML (v3.0) under the Jones-Taylor-Thornton (JTT) amino acid substitution model, with 100 replicates of bootstrap analysis, estimated proportion of invariable sites, four rate categories, estimated gamma distribution parameter, and optimized starting BIONJ tree [78], [79]. The phylogenetic trees were displayed using MEGA (v5.0) with 50% threshold of branch value [80].

### Chromosomal location and gene duplication

Genes were mapped on chromosomes by identifying their chromosomal position provided in the Phytozome database. Identification of segmental duplications resulting from salicoid genome-wide duplications was accomplished based on duplication coordinates from the *Populus* genome assembly v2.1. Blocks in the same colors represent the homeologous chromosomal segments.

To search for retrotransposons, BioMart online server at Phytozome website (http://www.phytozome.net/) was used to extract the flanking genomic sequences of 10-kb upstream and downstream of each HD-ZIP gene. Sequences were subjected to BLASTX searches against the GenBank non-redundant protein database at National Center for Biotechnology Information (NCBI) using E-value cutoff set to 1e^−10^. The output file was manually inspected for the presence of transposable elements including retrotransposons (LTR or non-LTR-related) and transposon (MULE, CACTA, hAT, and Heliton).

### Calculation of Ka/Ks Values

Amino acid sequences from segmentally duplicated pairs were aligned first by Clustal X v1.83 and the aligned sequences were subsequently transferred into original cDNA sequences using the PAL2NAL program (http://www.bork.embl.de/pal2nal/) [81], which uses the CODEML program of PAML [82] to estimate synonymous (Ks) and nonsynonymous (Ka) substitution rates. Divergence time (T) was calculated using a synonymous mutation rate of λ substitutions per synonymous site per year as T = Ks/2λ (λ = 9.1×10^−9^ for *Populus*) [74].

### Gene structure analysis

The exon/intron organization for individual HD-ZIP gene was illustrated with Gene structure display server (GSDS) program (http://gsds.cbi.pku.edu.cn/) [83] by alignment of the cDNAs with their corresponding genomic DNA sequences from Phytozome (http://www.phytozome.net/poplar, release 2.1).

### Identification of conserved motifs

The program MEME (v4.3.0) (http://meme.sdsc.edu) was used for the elucidation of motifs in 63 deduced *Populus* HD-ZIP protein sequences. The following parameters were used: number of repetitions - any, maximum number of motifs - 20, and the optimum motif widths were constrained to between 6 and 200 residues. Structural motif annotation was performed using the SMART (http://smart.embl-heidelberg.de) and Pfam (http://pfam.sanger.ac.uk) databases.

### EST profiling and microarray analysis

The expression profile for each gene was obtained by evaluating its EST representation among 19 cDNA libraries derived from different tissues and/or developmental stages available at PopGenIE (http://www.popgenie.org/) [75]. The heatmap was visualized using Heatmapper Plus tool at the Bio-Array Resource for Plant Functional Genomics (http://bar.utoronto.ca/ntools/cgi-bin/ntools_heatmapper_plus.cgi) [84].

The microarray data for various tissues/organs and developmental stages available at NCBI Gene Expression Omnibus (GEO) database [77] under the series accession numbers GSE13990 and GSE13043 were used for the tissue-specific expression analysis. The series GSE13990 includes Affymetrix microarray data from nine different tissue samples representing three biological replicates [61], whereas series GSE13043 contains NimbleGen microarray data from five stem internodes from the apical bud to the base of the shoot (internode 2 to internode 5, and internode 9) in two biological replicates [76]. For Affymetrix microarray data GSE13990, the Affymetrix CEL files representing nine tissues/organs as well as photoperiodic treatments were downloaded from GEO database at NCBI and imported into GeneSpring GX (V11.5) software (Agilent Technologies) for further analysis. The data was normalized by the Gene Chip Robust Multiarray Analysis (GCRMA) algorithm followed by log transformation and average calculation. After normalization and log transformation of data for all the *Populus* genes present on the chip, the log signal intensity values for *Populus* probe IDs corresponding to HD-ZIP gene model (v1.1) (Table 1 and Table S5) were extracted as a subset for further analyses. The tab-delimited files for the average log signal intensity values were imported into Genesis program (v1.75) to generate heatmaps [85]. Hierarchical clustering was performed based on Pearson coefficients with average linkage rule. NimbleGen array data GSE13043 were normalized using the NimbleGen microarray data processing pipeline (NMPP) [86]. Each gene model is represented by three replicated 60 mer isothermal probes on the array. Background hybridization intensity for determining expressed genes was estimated using signal intensity of negative control probes on the array. Gene-level analyses were performed using the mean normalized fluorescence values for all probes and replicates. The log signal intensity values for *Populus* probe IDs corresponding to HD-ZIP gene model (v1.1) (Table 1 and Table S5) were extracted as a subset for further analyses. The tab-delimited files for the average log signal intensity values were imported into Genesis program (v1.75) to generate heatmaps [85]. Clustering of gene expression was performed using hierarchical algorithm based on Pearson correlations.

For abiotic and hormone treatments, Affymetrix microarray data available at NCBI GEO database under the series accession numbers GSE17230 (drought stress) and GSE17686 were analyzed [87], [88]. GSE17686 is composed of the following five subset series: GSE14893 (nitrogen limitation, genotype 1979), GSE14515 (nitrogen limitation, genotype 3200), GSE16783 (one week after leaf wounding), GSE16785 (90 hours after leaf wounding) and GSE16773 (methyl jasmonate-elicited suspension cell cultures). The Affymetrix CEL files representing different abiotic and hormone treatments were downloaded from GEO database at NCBI and preprocessed by using GeneSpring GX (V11.5) software (Agilent Technologies). The data was normalized by GCRMA algorithm followed by log transformation and average calculation. After normalization and log transformation of data for all the *Populus* genes present on the chip, the log signal intensity values for *Populus* probe IDs corresponding to HD-ZIP gene model (v1.1) (Table 1 and Table S5) were extracted as a subset for further analyses. Expression was indicated as fold change of experimental treatments relative to control samples. The tab-delimited files for the average log signal intensity values were imported into Genesis program (v1.75) to generate heatmaps [85]. Hierarchical clustering was performed based on Pearson coefficients with average linkage rule.

Probe sets corresponding to HD-ZIP genes were identified using an online Probe Match tool available at POParray (http://aspendb.uga.edu/poparray). For probe sets matching several *Populus* HD-ZIP gene models, only those exhibited the highest hybridization signals consistently across multiple samples were considered. The list of probe sets corresponding to *Populus* HD-ZIP genes was provided in Table S5.

### Plant material and growth conditions

Plant material was collected from clonally propagated one-year-old *Populus deltoides* grown in the growth camber under long day conditions (16 h light/8 h dark) at 25–28°C. Shoot tip from stem internodes 1–3, young leaves from stem internodes 4–6, and root tissues were separately collected. Bark with phloem attached from the basal internodes was peeled off of the stem, phloem was scraped from the inside of the bark, and developing xylem was scraped from the outer layers of the wood. All samples were immediately frozen in liquid nitrogen and stored at −80°C until RNA isolation.

The clonally propagated six-month-old Nanlin 895 (*Populus×euramericana cv*) plants were used in stress treatments. Salt stress was conducted by watering plants with sodium chloride (NaCl) solution at concentration of 200 mM to saturation. For drought treatment, the intact root systems of plants were removed from the pots, washed gently with water to remove soil and then laid down on filter paper with 70–80% humility at 25°C under dime light. Two biological replicates were performed for each stress treatment. After exposure to stresses after 0, 1, 3, 6, 9, 12, and 24 hours, young leaves from three different plants were harvested at various time points, flash frozen in liquid nitrogen, and stored at −80°C for further analysis.

### RNA isolation and qRT-PCR

Total RNA from shoot tip, leaf, differentiating xylem, and phloem was extracted using TRIzol reagent (Invitrogen, Ca, USA) according to manufacturer's instructions. Alternatively, total RNA from bark and roots was isolated by CTAB method with minor modifications [89]. RNA integrity was verified by 2% agar gel electrophoresis. Before cDNA synthesis, RNA was treated with RQ1 RNase-free DNase (Promega, Madison, WI, USA) according to the manufacturer's instructions to ensure no DNA contamination, and then the first-strand cDNA synthesis was carried out with approximately 2 µg RNA using the RevertAid First Strand cDNA Synthesis Kit (MBI, Fermentas) and oilgo-dT primers according to the manufacturer's procedure. Primers were designed using Beacon Designer v7.0 (Premier Biosoft International, California, USA) with melting temperatures 58–60°C, primer lengths 20–25 bp and amplicon lengths 50–200 bp. All the primer sequences were listed in Table S6.

qRT-PCR was conducted on LightCycler® 480 Detection System (Roche, Penzberg, Germany) using SYBR Premix Ex Taq (TaKaRa, Toyoto, Japan). Reactions were prepared in a total volume of 20 µl containing: 10 µl of 2×SYBR Premix, 2 µl of cDNA template, 0.4 µl of each specific primer to a final concentration of 200 nM. The reactions were performed as the following conditions: initial denaturation step of 95°C for 10 s followed by two-step thermal cycling profile of denaturation at 95°C for 5 s, and combined primer annealing/extension at 60°C for 1 min for 40 cycles. No-template controls were included for each primer pair and each PCR reaction was performed in triplicate. To verify the specificity of the amplicon for each primer pair, a melting curve analysis was performed ranging from 60°C to 95°C with temperature increasing steps of 0.06°C/s (5 acquisitions per °C) at the end of each run. Baseline and threshold cycles (Ct) were automatically determined using the LightCycler® 480 Software release 1.5.0. Relative expression was calculated by the ΔΔCt method [90] using the geometric mean of two reference genes: *UBQ10* and *UKN1*(*Populus* orthologue of *Arabidopsis AT4G33380*) for different tissues, *UBQ10* and *PP2a* for abiotic stress treatments. The normalization factor (NF) of two reference genes was calculated and the relative abundance of target genes was analyzed using the geNorm (V3.5) software package [91].

## Supporting Information

Figure S1 **Exon/intron organization of** ***Populus*** **HD-ZIP genes.** Exons and introns are represented by green boxes and black lines, respectively. The numbers indicate the splicing phases of the HD-ZIP genes, 0 refers to phase 0, 1 to phase 1, and 2 to phase 2.
(EPS)

Table S1 **A complete list of** ***Populus*** **HD-ZIP gene sequences identified in the present study.** The list comprises of 63 HD-ZIP sequences identified in this study. Amino acid sequences were deduced from their corresponding coding sequences and genomic DNA sequences were obtained from Phytozome (http://www.phytozome.net/poplar, release 2.1).
(XLS)

Table S2 **A list of HD-ZIP protein sequences identified from eight plant species in this study.**
(TXT)

Table S3 **Pairwise identities between paralogous pairs of HD-ZIP genes from** ***Populus*** **.** Pairwise identities and sequence alignments of the 28 paralogous pairs identified from *Populus* HD-ZIP gene family.
(XLS)

Table S4 **Sequence logos for the conserved motifs of** ***Populus*** **HD-ZIP proteins.** Conserved motifs and the sequence logos were generated using the MEME search tool. Numbers on the horizontal axis represent the sequence positions in the motifs and the vertical axis represents the information content measured in bits. Motif 1 and 2 represents the homeodomain (HD), motif 7 represents the Leucine-Zip domain (LZ), motif 3 represents the START domain, motif 13 and 19 represents MEKHLA, and motif 18 represents the CPSCE.
(XLS)

Table S5 **A list of probes corresponding to** ***Populus*** **HD-ZIP for microarray analysis.**
(XLS)

Table S6 **A list of primer sequences of the 16 selected HD-ZIP genes for qRT-PCR analysis.**
(XLS)
